# Meta-consent for the secondary use of health data within a learning health system: a qualitative study of the public’s perspective

**DOI:** 10.1186/s12910-021-00647-x

**Published:** 2021-06-29

**Authors:** Annabelle Cumyn, Adrien Barton, Roxanne Dault, Nissrine Safa, Anne-Marie Cloutier, Jean-François Ethier

**Affiliations:** 1 Groupe de Recherche Interdisciplinaire en Informatique de la Santé (GRIIS), Faculté de Médecine et des Sciences de la Santé, Université de Sherbrooke, 2500 Boulevard de l’Université, Sherbrooke, QC J1K 2R1 Canada; 2 Centre National de la Recherche Scientifique (CNRS) - Institut de Recherche en Informatique de Toulouse (IRIT), 29 rue Jeanne Marvig, 31055 Toulouse, France

**Keywords:** Learning healthcare system, Meta-consent, Health data, Public opinion, Focus groups

## Abstract

**Background:**

The advent of learning healthcare systems (LHSs) raises an important implementation challenge concerning how to request and manage consent to support secondary use of data in learning cycles, particularly research activities. Current consent models in Quebec were not established with the context of LHSs in mind and do not support the agility and transparency required to obtain consent from all involved, especially the citizens. Therefore, a new approach to consent is needed. Previous work identified the meta-consent model as a promising alternative to fulfill the requirements of LHSs, particularly large-scale deployments. We elicited the public’s attitude toward the meta-consent model to evaluate if the model could be understood by the citizens and would be deemed acceptable to prepare for its possible implementation in Quebec.

**Methods:**

Eight focus groups, with a total of 63 members of the general public from various backgrounds were conducted in Quebec, Canada, in 2019. Explicit attention was given to literacy levels, language spoken at home and rural vs urban settings. We assessed attitudes, concerns and facilitators regarding key components of the meta-consent model: predefined categories to personalized consent requests, a dynamic web-based infrastructure to record meta-consent, and default settings. To analyse the discussions, a thematic content analysis was performed using a qualitative software.

**Results:**

Our findings showed that participants were supportive of this new approach of consent as it promotes transparency and offers autonomy for the management of their health data. Key facilitators were identified to be considered in the implementation of a meta-consent model in the Quebec LHSs: information and transparency, awareness campaigns, development of educational tools, collaboration of front-line healthcare professionals, default settings deemed acceptable by the society as well as close partnerships with recognized and trusted institutions.

**Conclusions:**

This qualitative study reveals the openness of a sample of the Quebec population regarding the meta-consent model for secondary use of health data for research. This first exploratory study conducted with the public is an important step in guiding decision-makers in the next phases of implementing the various strategies to support access and use of health data in Quebec.

**Supplementary Information:**

The online version contains supplementary material available at 10.1186/s12910-021-00647-x.

## Background

Within learning health systems (LHSs), people’s health data are used on a large scale to generate new knowledge that will be quickly applied in clinical practice to support continuing improvement in the quality care [1]. Over the last decade, LHSs have demonstrated their success in various settings and contexts by ensuring innovation, securing quality and safety, as well as achieving the objective of improving health care delivery in a continuous fashion [2, 3].With that in mind, the province of Quebec (Canada) is currently working on the implementation of a learning data system named “PARS3” to support, among other things, the secondary use of primary care health data for research [4]. However, one of the major issues related to access and use of people’s health data in this new context of research is the request and management of consent.

The current consent models in Quebec regarding the use of people’s health data for research purposes were not established with LHSs in mind. The standard project-specific individual consent is impractical on such a scale and severely hinders deployment of important projects. An implementation of delegated consent for access to health data for research has been made possible in hospital contexts under the *Act respecting Access to documents held by public bodies and the Protection of personal information* (CQLR c A-2.1, s 125) of the Compilation of Québec Laws and Regulations. However, it does not promote transparency—and therefore autonomy—optimally, as it does not inform healthcare users about the use of their health data. In addition, it does not provide access to health care data distributed outside of the hospital context.

As has been empirically confirmed by a provincial survey conducted in 2018 [5], Quebec citizens largely support the use of people’s health data for research to advance knowledge and improve care, but mostly disagree with the current model of delegated consent for secondary use of their health record data. They greatly value transparency and consent: most of them want to be informed and to be asked for consent for the use of their health data for research.

A new approach to consent for the use of health data is needed with the advent of LHSs in Quebec. A literature review performed in 2018 provided a broad assessment of published approaches of consent in LHSs contexts [6]. One solution stands out to meet the challenges of consent for data access in a LHS context: the meta-consent model proposed by Ploug and Holm [7]. It is specifically adapted to the requirements of LHSs and could overcome many of the obstacles with the current consent models including operability on a large scale. The basic idea of the meta-consent model is that people are being asked to design how and when, in the future, they would like to provide consent to the use of their personal health data [7]. This approach gives the patient an active role within the consent process in addition to favouring transparency regarding data use. It is arguably practical, respectful, and supportive of participant autonomy [7]. Inspired by this approach, we asked in a population-based survey in Quebec how citizens would feel about a web-based portal that would allow them to be notified of the use of their health data for upcoming research projects and to express their consent preferences on this matter (along the lines of meta-consent) [5]. The vast majority of them found it important that such a web-based portal would be created. The survey confirms the expectation of the Quebec public to be involved in the management of their health data in research.

## Methods

### Study design

As part of a sequential mixed methods study, a qualitative approach consisting of semi-structured focus groups was used to explore Quebec citizens’ perspectives regarding the use of health data for research purposes and specific elements of the meta-consent model required to meet the unique particularities of a LHS. These specific elements include: predefined categories to personalized consent requests, an infrastructure to request and record meta-consent as well as modifiable and immutable default settings. In addition to open-ended questions, practical exercises were conducted with the participants to ensure deeper understanding regarding the meta-consent framework and support discussions (see Additional file 1: Tables S1.5–S1.6).

### Participants selection and recruitment

Focus group participants were selected from a panel of 30,000 Quebec citizens owned by BIP Research polling firm [8]. This panel consists of people recruited over the years following telephone interviews and voluntary registrations on the firm’s website. Using information from their socio-demographic profile, potential participants were identified from this panel and contacted by phone by trained interviewers to assessed eligibility and interest to participate in this study. To be part of one of the focus groups, participants needed to be Quebec residents, 18 years or older, speak and understand French or English, have a minimum literacy threshold ascertained by asking two questions formulated using the International Assessment of Adult Competencies proficiency levels of 2012 (level 1) [9], and be available to participate in person in a 2 h focus group at a set date, time and location in their region. Participants were given a monetary compensation for their participation in the study (100$ CAN voucher).

### Focus groups’ procedure

The discussion guide and the tools used in the focus groups were based on prior literature [6], findings from the previous phase of this study [5], and recurrent input from a patient-partner (LDP) and two non-profit literacy organizations in Quebec [10, 11]. The discussion guide and the tools were piloted with three groups to assess comprehension, fluidity in the process and logistical details (e.g., duration). Following these pilot groups, several modifications were made to the discussion guide and the tools to meet a low literacy level.

Eight semi-structured focus groups were conducted across the province of Quebec between May and June 2019, consisting of 6 to 11 participants and lasting approximately 120 min for 2 h. Each focus group was moderated by an experienced moderator (NS) accompanied by a note-taker (RD) and was video-recorded.

#### Focus groups’ composition

The eight focus groups were formed based on three criteria: language with greatest fluency, primary area of residence, and level of education. Our goal in creating homogenous groups was to facilitate spontaneous discussions and enhance group dynamics while covering a broad range of backgrounds that could influence participant’s views on the topic [12]. Because education level is associated with health status, digital literacy, and health-information literacy [13], it was used as a proxy for the literacy level. Half of the groups were conducted with participants with a completed high school diploma or higher education (≥ HSD), while the other half of the groups included participants who did non-complete high school (No HSD). We conducted six focus groups in French and two focus groups in English in four locations across the Quebec province: Sherbrooke, Quebec City, Rimouski and Montreal. The composition of each group is presented in Additional file 1: Table S1.1.

Since participants had various levels of knowledge concerning research involving health data, we developed two short animated video for the purpose of this study [14]: a first one explaining the nature of health data and how they might be used by researchers to contribute to the improvement of care, and a second one which briefly introduced the meta-consent model by presenting the idea of a web-based portal on which people could dynamically customize their consent preferences for the use of their health data according to different categories of research projects. Open discussion periods were added at the end of each video during which the moderator gathered participants’ attitude and questions. Afterward, the moderator discussed further with the participants about specific elements of the meta-consent model using a visual presentation, in order to explore the attitude of participants towards this model as well as the major concerns and facilitators regarding its implementation in Quebec.

Three aspects of the meta-consent model were explored with our groups: the comprehension of the approach using exemplar predefined categories to personalized consent requests, the importance and the characteristics of a web-based infrastructure to request and record meta-consent preferences as well as acceptability of modifiable and immutable default settings.

#### Comprehension using predefined categories to personalized consent requests

For the purpose of this study, we needed to concretize the meta-consent model for the participants to evaluate the comprehension of the approach and its acceptability. We used two data content characteristics to illustrate important aspects that can modulate the choice of consent: (X) data identification character (with vs. without direct identifiers), genetic data usage (with vs. without genetic information); and two contextual characteristics: (Y) the type of organisation who has access to the data (academic or public vs. private organisation with commercial goals) and where could the data be used (only within Canadian borders vs. international). The model also includes four possible consent choices (Z): broad approval, specific opt-out, specific opt-in and broad refusal. These were determined based on Ploug & Holm’s original model [7], key findings of our scoping review [6], and consultations with a group of experts from various domains (ethics, information security, bioinformatics, health professionals, and patient-partners). The details of our meta-consent model are presented in Additional file 1: Tables S1.2–S1.4.

From these, we created categories of research projects, which were defined as a set of projects that have similar characteristics regarding the type data content required (X) and the context in which the research is conducted (Y), for example: without direct identifier, without genetic information, academic organisation, and international use. The four characteristics being binary choices, we had a total of 2^4^ = 16 categories. For each category of research projects, participants needed to choose a type of consent (Z) according to their preferences.

We explored the motivations underlying participants’ consent preferences for the use of their health data according to these 16 different combinations of research project characteristics. We opted for an approach by categories each defined by a combination of four binary characteristics, rather than an approach where the characteristics are assessed individually, in order to take into consideration the interaction between characteristics. We distributed a notebook to each participant in order to collect their individual answers (see Additional file 1: Tables S1.5–S1.6).

#### Attitudes towards an infrastructure to request and record meta-consent

We proposed a meta-consent framework implemented by an integrated dynamic web-based portal that generates meta-consent requests and reminders, allows individuals to record and modify their meta-consent parameters, and generates consent requests based on these parameters [7]. In the focus groups, we explored the general attitude and interest toward such a portal as well as the concerns, essential conditions and facilitators for its implementation in Quebec.

#### Attitudes towards modifiable and immutable meta-consent default settings

Some individuals might choose not to interact with the meta-consent infrastructure. To avoid blocking LHS activities, a meta-consent system needs to define a set of modifiable default settings that are implemented if a person does not provide his full meta-consent preferences (by explicitly accepting proposed baseline settings or by modifying them). Such defaults are a standard example of nudges, namely interventions on the context in which people make decisions with the aim of steering people’s behaviour into specific directions, while maintaining their freedom of choice [15, 16]. Some of these default settings might pertain to specific uses of health data as currently permitted by the law (e.g. access to record-based health data for retrospective research) [7]. Such default settings would be modifiable, that is, they would hold without any interaction of the user with the system, but the user could change this setting at any given time.

Ploug & Holm propose that setting a default of broad consent strikes the right balance between the different interests at stake. We have therefore explored this proposal with our participants. We tried to elicit a) participants’ attitude towards the idea of modifiable default settings and b) facilitators regarding the acceptance of a modifiable broad consent default setting (BCDS), as well as c) categories of research projects for which a modifiable BCDS would be acceptable and those for which it would not be acceptable.

We also explored “immutable” default settings with our participants, namely baseline settings that cannot be changed by the citizens. These immutable default settings could be set to a broad approval, specific opt-out, specific opt-in or potentially even a broad refusal for certain categories of research projects. Immutable BCDS are similar to the “obligation to share”. For example, Quebec’s public health is legally allowed to have access to citizens’ health data in some circumstances (e.g. for the management of health emergencies such as the COVID-19 crisis). We were therefore interested in exploring the perception of participants of such an obligation to share, depending on the context of use. We were interested in their view regarding the contexts for which it would be acceptable to have immutable settings that allow sharing of data by default (that is, an immutable broad consent default setting).

### Data analysis

All video-recorded focus groups were transcribed into verbatims and imported into the qualitative data analysis software NVivo version 11 pro (QSR International). Field notes, including facial and non-verbal cues, were added to the verbatims. A list of nodes (i.e. a code, theme, or idea) was generated a priori using the themes covered in the discussion guide. Additional nodes were created during coding as new themes emerged from the dataset and were validated with the research team to ensure their relevance. Each node was defined using Aristotelian definitions (a good practice in writing definitions) to ensure accuracy of coding [17]. The two first focus groups were coded and simultaneously discussed together by the two coders (RD and NS) and entirely reviewed by a third research member (AB). The six other focus groups were then coded independently by the two coders (RD and NS). All coding discrepancies were resolved through discussion until a consensus was reached. When consensus was not reached, a third reviewer was involved (AB). Using an iterative process, the research team met to discuss the coding of the transcripts periodically. We established that data saturation was obtained when no new themes or codes emerged from the dataset.

## Results

Table 1 presents the characteristics of our focus group participants. Overall, 63 Quebec citizens took part in our eight focus groups. Participants were from various age groups (20 to 70 years old), were infrequent users of the healthcare system (62% with less than three healthcare visits in the last year) and were not very familiar with health research (only 16% previously participated in health research). A great majority of them had a family doctor (83%) and were regular internet users (89% use internet at least once a day).

**Table 1:** Focus group participants’ characteristics

	All participants (n = 63)	No HSD (FG 1-3-5–7) (n = 29)	≥ HSD (FG 2-4-6-8) (n = 34)
*Gender*			
Female	32 (50.8%)	12 (41.4%)	20 (58.8%)
Male	31 (49.2%)	17 (58.6%)	14 (41.2%)
*Age—median (range)*	50 (20–70)	51 (27–70)	47 (20–70)
*Education*			
No HSD	27 (42.9%)	29 (100%)	0
HSD or equivalent	6 (9.5%)	0	5 (14.7%)
Diploma of Collegial Studies	13 (20.6%)	0	12 (35.3%)
University diploma or equivalent	17 (27.0%)	0	17 (50.0%)
General health*			
Excellent	10 (15.9%)	4 (13.8%)	6 (17.6%)
Very good	26 (41.3%)	9 (31.0%)	17 (50.0%)
Good	20 (31.7%)	11 (37.9%)	9 (26.5%)
Fair	6 (9.5%)	4 (13.8%)	2 (5.9%)
Poor	1 (1.6%)	1 (3.4%)	0
*Chronic disease—yes*	21 (33.3%)	13 (44.8%)	8 (23.5%)
*Family doctor—yes*	52 (82.5%)	21 (72.4%)	31 (91.2%)
*Healthcare visits in the last 12 months*			
0 time	9 (14.3%)	6 (20.7%)	3 (8.8%)
1–2 times	30 (47.6%)	12 (41.4%)	18 (52.9%)
3 + times	24 (38.1%)	11 (37.9%)	13 (38.2%)
*Previous participation in health research—yes*	10 (15.9%)	3 (10.3%)	7 (20.6%)
*Internet at home/on cell phone—yes*	61 (96.8%)	28 (96.6%)	33 (97.1%)
*Frequency of Internet use*			
At least one time/day	56 (88.9%)	23 (79.3%)	33 (97.1%)
At least one time/week	5 (7.9%)	4 (13.8%)	1 (2.9%)
Rarely	2 (3.2%)	2 (6.9%)	0
Never	0	0	0

HSD: High School Diploma

*Self-reported health using the single question from the SF-36 Health Survey and the International Quality of Life Assessment: “In general, would you say that your health is excellent, very good, good, fair, or poor?” [18]

Below, the qualitative results are presented according to the themes described above. More specifically, for each theme, we report participants’ general attitudes as well as the main concerns and facilitators.

### Secondary use of people’s health data for research purposes

#### General attitude

Participants overwhelmingly agreed that people’s health data should be used in research for the purpose of improving medical practice and care:

“As far as my medical records are used to advance people’s health care, I’m all for it. I honestly wish certain data had been released 50 years ago.” (male, no HSD, 50–59 years old)

Some participants acknowledged that they or their family members had benefited from prior research, and this particularly motivated them to contribute to research with their own data for the benefit of others.

However, participants expressed a strong opinion in favour of autonomy in the sense of freedom of choice. A participant even raised the issue of data ownership. According to her, these data belong to the citizens which is why it is imperative that individuals have the right to control the use of their information and to decide whether to share them or not, which is a good illustration of an opting choice:

“Does that information belong to me? And if so, do I have the right to say: ask me and if I don’t answer it’s because I refuse?” (female, ≥ HSD, 50–59 years old)

#### Concerns

Participants expressed several concerns regarding the use of health data for research purposes and regarding the implementation of an electronic system to support such use. It is important to note that these concerns were largely associated with a lack of trust in certain stakeholders and fear regarding unauthorized access to personal information in general. The following concerns were raised by participants:

##### Security breach and unauthorized access by third parties

There were concerns about the security of personal data due to flaws in computer systems, generating a lack of trust. These fears seemed to be largely a reflection of a more general fear about the security of personal data:

“Some people are more cautious about their personal data and are afraid about security breaches in the system. We know there are security breaches everywhere, especially regarding people’s identities.” (female, ≥HSD, 50–59 years old).

Some participants noted, however, that the risk of security breaches regarding access to health data already exists, regardless of the format of the data or their use for research purposes:

“Our medical file is already computerized. So, there is already a risk that someone might using it wrong. I don’t think there is much more risk than the one that already exists.” (male, ≥HSD, 30–39 years old)

Specific concerns were raised regarding the access and use of people’s health data by unauthorized third parties, such as employers or insurance companies. These fears were particularly strong regarding sensitive data, such as data related to mental health and sexually transmitted and blood-borne diseases, as it can affect the coverage status or the ability of an individual to get a job:

*“*If someone wants a job and, because of security breaches in the system, the employer could access his medical records to see if he has certain conditions, this person might not be hired.” (male, no HSD, 60–69 years old).

“Those who are really interested in this are the insurance companies to tell you ‘I don’t insure you anymore’ or ‘your insurance will increase’.” (male, ≥ HSD, 50–59 years old).

##### Private research with commercial purposes

Many participants were also reluctant to allow health data to be used by private companies for commercial and profit-making purposes. There was also a perception by some participants that the private sector has fewer restrictions regarding data security and the ethics of handling data:

“The more we go to the private sector, the more freedom they have. They can do what they want with the data. They could contact the insurance companies, sell the data...We don’t know.” (male, no HSD, 50–59 years old)

Some participants acknowledged, however, that private research can bring health benefits:

“[…] we know that even if it’s private, it doesn’t mean that they won’t find something interesting. They can also help people with their research.” (male, no HSD, 60–69 years old)

##### Uncertainties regarding data use in the future

Acknowledging evolution of technology and law over time, some participants expressed fear regarding the use of their health data in the future:

“What can they do with it? Well, today they may not be able to do anything with it, but who knows if in 10–15 years maybe they will be... I don't want to live in fear, but that’s why I would like to know who have access to my data and why.” (male, no HSD, 40–49 years old).

#### Facilitators regarding data sharing

##### Transparency

Transparency about who is using the data, what data will be used, and for what purposes was, by far, a fundamental prerequisite regarding the use of people’s health data for research. Transparency alleviated many of the fears outlined above.

“I want to be advised that they are looking into and go into my records. I will give you my consent because I know what you’re going to go looking for.” (female, no HSD, 50–59 years old).

For many participants, transparency was even more important than the consent process itself.

##### Confidentiality

It was important for the majority of participants that the data should not allow direct identification (names) or to contact them directly (e.g., phone number, postal address). The concern to be identified through health data appeared less present for participants who were overall healthy:

“I don’t care, I have nothing to hide.” (male, no HSD, 30–39 years old)

### Meta-consent process and preferences

Throughout the practical exercises, all participants, regardless of their age and their education level, showed good understanding of the core concepts of the meta-consent model. Supported by short explanations and concrete examples, participants were easily able to express and explain their consent choices regarding the predefined categories of research projects that we had presented to them.

Participants greatly appreciated the dynamic aspect of the model (that is, the possibility of changing their meta-consent choices over time) as well as the availability of a spectrum of consent options (four types of consent rather than just “yes” or “no”). They particularly endorsed the specific opt-in consent and specific opt-out consent, as they promote autonomy and freedom of choice. These two types of consent were very reassuring for many participants as they saw them as a way of making more informed decisions regarding the use of their data for research:

“I would like to be asked because if I think it’s important and it can help some sick people to be healed, yes, there’s no problem. But if I see that it’s not relevant and that it could be a bit of anything, then I might refuse.” (male, no HSD, 20–29 years old)

**Fig. 1:**
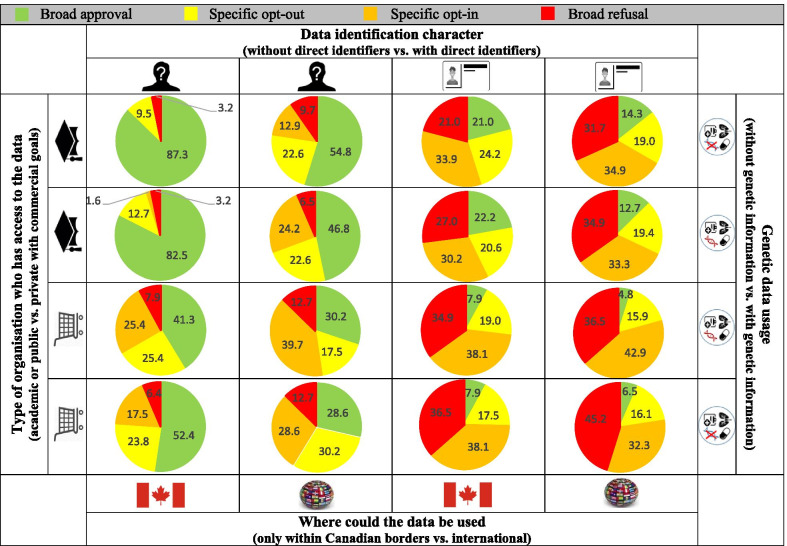
Participants’ meta-consent preferences for the 16 predefined categories of research projects. *Note*: This figure was made by author Roxanne Dault

Participants’ choice of consent for each predefined category of research projects (the 16 combinations of the four binary characteristics) are presented in Fig. 1. From these results, it is possible to observe trends regarding certain categories of research projects. For example, 87% of the participants chose a broad approval for the following combination of characteristics: data without direct identifiers, without genetic information, accessed by a public/academic organisation, and used at the national level only. For other categories of research projects, we noted a high degree of variation in consent choices between participants, showing that there are significant differences in consent preferences within the population tested. Analysis of comments indicates that this variation can be explained by various underlying reasons, including personal values and perception of risk. Some characteristics had a more pivotal impact on the participants’ choice of consent. The presence or absence of direct identifiers was the most cited characteristic by the participants to affect their choice of consent within a given research category. We could observe a tendency towards higher proportions of “broad refusal” and “specific opt-in” choices when the following characteristics were present (alone or combined in a given category of research projects): direct identifiers, private organisations with commercial goals, and international scale of use. Additional file 2 presents several quotes from focus groups’ participants regarding the reasons underlying the consent choices as well as fears and reassuring factors raised for each of the eight characteristics that compose the different categories of research projects.

[A participant who choose a broad refusal for the following category of research projects: data with direct identifiers; with genetic information; accessed by a private organisation with commercial goals and used at an international level] “I refuse because I don't want my identity to be seen. It [the choice of consent] is more about the identity.”

### A web-based infrastructure to request and record meta-consent

#### General attitude

On a personal level, participants had mixed interest in a web-based consent portal on which they could dynamically record their meta-consent preferences and authorize the use of their health data for research. Some participants saw it as a good way to take control over their own data and ensuring transparency in the process of access and use by the stakeholders, resulting in a feeling of reassurance:

“For me, it [consent portal]’s a must because it’s kind of a control thing. I would be able to see who’s using it and why.” (female, ≥ HSD, 40–49 years old)

Participants who were personally affected by chronic or rare conditions, or had loved ones affected by such conditions, were especially interested in being informed about new researches on such conditions and having access to a consent portal:

“I have people in my family who have multiple sclerosis. If they have this [consent portal], they’ll definitely go to use it right away.” (male, ≥ HSD, 30–39 years old)

However, some other participants did not express the desire to directly use such a tool or had some reservations. Reasons mentioned were the preference to delegate such decisions, a lack of interest, usage-induced anxiety and digital divide.

##### Preference to delegate

Some participants mentioned that they would prefer to delegate their choice to someone they trust such as the family physician, an accessible resource with good knowledge of their patients’ health and best interests:

“My doctor knows my health problems and will be able to go into the computer and determine the right choice. He is already governed by the ethical code of his professional order.” (male, ≥HSD, 40–49 years old)

##### Lack of interest

Several participants mentioned that they have no time or desire to be involved in a complex process and would prefer that it would be a blanket consent^1^:

“I honestly don’t care. I wouldn’t go at all [on the consent portal]. I have better things to do than deal with this. Let them take it [my health data] and do what they want with it.” (male, ≥HSD, 30–39 years old)

Others had particular difficulty perceiving clear or direct benefits of using this type of consent portal for themselves or for their family, nor any negative personal consequences of not using it:

“When we try to pay an electricity bill, we have no choice... But in this case [record my consent preferences], I won’t be in trouble at the end of the month and there is no one who is going to bother me if I haven’t done it.” (male, ≥HSD, 30–39 years old)

##### Usage-induced anxiety

A concern that using this consent portal might provoke anxiety, because of sensitive information that it would contain and, because of a fear of over-solicitation, mainly regarding the numerous portal queries to participate in research projects:

“Checking out the portal would just make me uncomfortable seeing what is negative.” (female, ≥ HSD, 20–29 years old)

“What does the quantity look like? I mean, if we are getting 10 emails a day, we might get annoyed” (male, ≥ HSD, 30–39 years old)

##### Digital divide

A practical concern was raised regarding the social inequalities associated with the access and use of an informatic tool. Many participants, particularly in the low education groups, pointed out the difficulties regarding Internet access and the opportunity or knowledge to use computers (which has been called the “digital divide” by Steele 2019 [20]), as well as the ability to understand the consent portal itself and the information presented on it:

“I think it’s going to be a little scary. Not everyone is that good with computers. Like me, I hate computers. I think that is something that will drive people away.” (male, no HSD, 60–69 years old)

Some participants noted that elderly or individuals who suffer from certain mental conditions may have particular difficulties in using the portal, whereas the data of such participants may be particularly relevant for research.

#### Facilitators regarding the use of the web-based consent portal

A few factors were raised by participants that, in their opinion, could increase the population’s level of acceptability towards such a web-based portal for meta-consent.

##### Support from healthcare professionals

Many saw healthcare professionals, especially family physicians, as key resources in supporting citizens for the use of the consent portal. This support was generally envisioned in two ways: As being a credible and trusted source to inform people about the existence of the portal and the importance of its use, and as having a supporting role to better understand the choices proposed on the portal and in the decision-making process. This opinion seemed particularly pronounced in older and low-education participants:

“If I go see my doctor, I will talk to him about it [consent portal]. I’ll be more satisfied, because I can ask my questions. He will tell me in another way I could understand more.” (male, no HSD, 60–69 years old)

##### Awareness

Most participants particularly insisted on the need to be well-informed about this portal. In particular, they wanted to be informed about the existence of the consent portal and how to use it, and also to give the portal a period of trial:

“We need to give the program [consent portal] time to prove itself. Right now, everyone is reluctant, we don’t really know what it is. […] When it will be well implemented for a few years, well structured and all that, people will adhere more and more. You cannot turn the whole planet upside down with a snap of the finger.” (female, ≥HSD, 50–59 years old)

##### Anchored to a recognized institution

Several participants mentioned that anchoring the consent portal with a recognized institution would reinforce trust and ease of use. Participants suggested an institutional candidate such as the provincial health insurance (*Régie de l’assurance maladie du Québec* [RAMQ]) [21], which is already responsible for hosting the Québec Health Booklet [22], a provincial patient portal allowing access to health data online: it is already known, recognized and trusted by the citizens, and do not require the use of new access codes and passwords:

“I think it’s really important that the RAMQ has some kind of endorsement here. If I don’t see that organization sponsoring this [consent portal], I will not trust it.” (male, ≥HSD, 50–59 years old)

“Many people use The Québec Health Booklet portal already. The Quebec government is already advertising its benefits. So, if the consent portal was directly inside [it], we could go and easily make our choices.” (male, ≥HSD, 60–69 years old)

##### Alternatives to the informatic tool

Many participants pointed out that to avoid social inequalities, alternatives to electronic access to the portal must exist. People need to have the opportunity to record their meta-consent preferences through medias other than electronic devices, so that a variety of people are not excluded—e.g. people with literacy problems, restricted access to electronic tools, or restricted ability to use them.

### Meta-consent modifiable default settings

#### General attitude

Overall, participants agreed with the idea of modifiable meta-consent default settings that would hold for people who do not explicitly record their meta-consent preferences. A majority of the participants in the two groups (low education and high education) saw such defaults as providing a good balance between allowing research in realistic conditions and maintaining individual autonomy. What greatly favoured acceptability was the modifiable nature of the default setting which allowed people to have the control over the final decision to share or not their health data.

From the outset, several participants suggested that the use of people’s health data for research purposes should take the form of a broad consent, comparing it to the organ donation model in several countries (e.g., in Sweden). In their view, it should be the citizens’ responsibility to refuse the access to their health data if they do not agree with their use for research:

“I have been hearing more and more some countries around the world saying that the organs donation is by default ‘yes’ unless you put it ‘no’. At first, I was not sure but then the more I thought of it, well if there is a need and it’s really up to your personal accountability. Why isn’t ‘yes’ for everybody? By default, it’s all ‘yes’ unless you choose ‘no’.” (male, ≥HSD, 50–59 years old)

Despite the possibility to modify the default settings according to their preferences, a few participants deemed unacceptable to use people’s health data without their explicit consent. A participant specifically mentioned that we should focus instead on education and awareness in order to encourage people to express their consent preferences rather than adopting a uniform opt-out model.

#### Facilitators regarding modifiable broad consent default setting

##### Awareness campaigns

Participants strongly emphasized the need for information and awareness campaigns if a modifiable broad consent default setting (BCDS) is used for certain categories of research projects on the consent portal. The process must also be very transparent to avoid a public outcry:

“An awareness campaign, like people have to know. You know that the default is ‘yes’ then you have to go out to make sure that you opt-out. So, it has to be transparent and it has to be made clear and aware to the public.” (female, ≥HSD, 40–49 years old)

Many also believed that if a modifiable BCDS is used for certain categories of research projects, it would be better accepted by the public if there is a buffer period during which the portal information is not used, to leave some time for people to be adequately informed about this default setting and to change it if they want to. This buffer period would allow everyone to be adequately informed about the existence of the consent portal, as well as how to properly use it.

### Specific categories of research projects for which a modifiable BCDS would be acceptable

Many participants mentioned that they would agree with a modifiable BCDS, but only for specific categories of research projects. For that reason, we asked them to tell us for which categories they thought it would be acceptable among the 16 combinations of characteristics tested with them during the groups. The vast majority of participants found it acceptable to have a modifiable BCDS for the following category of research projects: “without direct identifiers, without genetic information, data accessed by an academic/public organisation and used within Canadian borders only”.

Upon analysis of the exchanges, it was clear that the characteristic the most strongly associated with an openness to a modifiable BCDS was the absence of direct identifiers (expressed as “anonymity” by most participants). For some participants, absence of direct identifiers alone was sufficient to accept a modifiable BCDS:

“I would put all ‘yes’ except for direct identifiers. That would be the basis. Everything ‘yes’, except the name” (female, no HSD, 30–39 years old)

Other participants expressed the need for data to be only accessible by public or academic organisations and only used within Canadian borders. Many participants expressed a clear discomfort with a modifiable BCDS for research conducted by private organisations with commercial goals and on an international scale, even in the absence of direct identifiers. Interestingly, the presence of genetic information did not appear to have a major impact on the decision regarding a modifiable BCDS for most participants.

### Meta-consent immutable default settings

#### General attitude towards immutable BCDS

Most participants found that immutable default settings allowing the use of health data for research might be acceptable, if the research is aligned with the population’s best interest and could lead to better care. Similar as for modifiable default settings, the participants mentioned that the acceptability of an immutable BCDS depends on the type of data shared and/or the context of data use (e.g., for sanitary emergency, public health):

“Let’s say it’s rare and very few people have this disease. It’s something scary to the population. That is why we would be obligated to give that information. We would want to know without their consent how did this person survived so we can save everybody else.” (male, no HSD, 30–39 years old)

A few participants, on the other hand, did not find the idea of an immutable default setting that allows the sharing of data acceptable in any way for several reasons. First, they feared that accepting immutable settings would result in a loss of control over their own health data:

“No one is going to say no if it’s for research that will lead to an important outcome. I’m not saying no because I’m negative, I’m saying no because I like to know what is going on. I also want to know what they are going to do with my data.” (female, ≥HSD, 60–69 years old)

Second, they were afraid of misuse of their information and the potential impact on privacy. There was a perception that an immutable BCDS would be associated with a loss of transparency from stakeholders. Finally, they perceived it as an infringement on their freedom of choice: many would accept to share their data for research, but however, want to maintain the final decision to share it or not.

#### Facilitators regarding immutable BCDS

Our discussion groups revealed several factors that would facilitate the acceptance of an immutable BCDS for some categories of research projects.

##### Transparency

Once again, transparency was the most important element for participants regarding the use of their health data, particularly in this particular context:

“I want to know exactly who has access. The exact name of the university. Why did that person have access to my data?” (male, ≥ HSD, 40–48 years old)

##### Confidentiality and accountability

Many mentioned that they would accept an immutable BCDS if stakeholders can prove that the confidentiality of their data is guaranteed. One participant added the necessity for accountability in case of security breaches compromising access to data:

“If I could be guaranteed complete anonymity, I would say yes. To have something in writing: the government signs off and says that he will protect your privacy.” (male, ≥HSD, 50–59 years old)

### Specific categories of research projects for which an immutable BCDS would be acceptable

We asked participants if it would be acceptable to set an immutable BCDS for the following category of research projects: “without direct identifiers, without genetic information, data accessed by an academic/public organisation and used within Canadian borders only”. Interestingly, almost all participants agreed across the eight focus groups:

“You don’t even have to ask the question, it [the data should be] available to the researchers.” (no HSD; unanimous agreement among the participants in this group)

### Who should be involved in decision making regarding the default settings for the society?

Several participants in various groups spontaneously mentioned that research ethics committees should be involved in decisions regarding the nature of both modifiable and immutable default settings that could be implemented in the consent portal. Many seemed to put great trust in research ethics committees and saw the combination of experts and citizen representatives as an asset to address this issue:

“The ethics committee because there is an accurate representation of citizens that have their own interests and everybody’s interest is represented.” (female, no HSD, 50–59 years old)

Participants also saw surveys and focus groups with key stakeholders (citizens, experts) as good ways to determine the modifiable and immutable default settings that should be implemented in the consent portal.

## Discussion

As part of a mixed-methods study, we conducted eight focus groups across the province of Quebec with two main objectives: to confirm the acceptability, for Quebecers, of the secondary use of health data for research purposes, and to explore attitudes, concerns, and facilitators regarding several features of the meta-consent model. Interesting findings emerge from the results: (1) confirmation of previous survey results regarding a strong support for secondary use of health data to improve patient care [5], (2) support for the meta-consent model as a credible alternative to current models of consent, (3) key facilitators to be considered in the implementation of a meta-consent model for the Quebec LHS. These facilitators include (a) ensuring information and transparency, (b) involving healthcare professionals, (c) establishing close partnerships with recognized institutions, (d) eliciting default settings deemed acceptable by the society, and (e) developing appropriate education tools and public awareness campaigns. All these elements were deemed central to foster trust and to engage and maintain citizens’ interest to share their health data for research. They also provide a first source of evidence in support of the meta-consent portal.

### Strong support for the secondary use of people’s health data for research to improve patients care

As observed in the quantitative phase of this mixed-methods study, Quebec citizens support the secondary use of people’s health data for research that aims at improving care to patients [5]*.* Across the literature, citizens and patients alike have expressed a willingness to contribute to research by sharing their health data for the purpose of advancing knowledge and improving patient care [23, 24]. Citizens also express a clear demand to be involved in the process. They named several conditions that needed to be respected for them to agree with the use of their health data for research, such as transparency, confidentiality and autonomy.

Our observations are consistent with the conclusions of a narrative review on patients’ and public views regarding the use of their health data for research purposes [24]. As well illustrated in this review, researchers and institutions need to address people’s diverse concerns and to invest efforts to meet the conditions identified. Without these conditions, institutions may lose trust which is vital for the support of healthcare and biomedical science. Results also support the idea that the public have concerns beyond legal compliance alone, and insist on the importance of information, transparency and control.

### The meta-consent model: a credible alternative to current consent models

Regardless of language, geographic location or level of education, the citizens who participated in our focus groups demonstrated a good understanding of the meta-consent model through practical exercises and open discussions. The findings from these exercises give legitimacy to a meta-consent model by evidencing that individuals have significantly different consent preferences depending on the characteristics of a research project (the type of data used, the identity of the data users, as well as the context in which the research is conducted).

These results echo those of a proof-of-concept study conducted by the initiators of the meta-consent model [25]. This study, conducted within the Danish population, identified similar themes: the consent choices made strongly indicate that there are significant differences in consent preferences within the population, the respondents felt that they understand the choices they were asked to make, and around twice as many want to be asked for specific consent for commercial research and for international research than for public research. The authors conclude that it is possible to ascertain meta consent preferences in the adult population by means of a mobile application. However, comparison of actual meta consent preferences must be made with caution: in order to take into account the interaction between the different characteristics of research projects, we assessed consent preferences for different categories of research projects defined by combinations of characteristics while Ploug and Holm assessed consent preferences for each characteristic individually [25].

The participants agreed that this consent model is a credible alternative to the current models including delegated consent for two main reasons: (a) the respect of autonomy, by providing people the possibility to control the future use of their health data in various contexts and, (b) the dynamic nature of the model, in which consent choices can evolve over time according to values, trust and vulnerabilities. This model’s emphasis on transparency and information was central to its acceptability.

Participants were very conscious of the challenges related to the web-based nature of the proposed approach. Implementation of a web-based meta-consent portal would require careful interface design to ensure that it is user-friendly and easily accessible and adapted for people with different levels of literacy, including digital literacy.

Furthermore, digital divide, known as the limited access to computers and Internet (a phenomenon which particularly concerns elderly, the disabled and individuals in socially deprived communities) also needs to be seriously considered and studied [26]. Recent experiences from the use of online portals for consent show that populations that enrol by an electronic route are less diverse in terms of ethnicity and education than populations enrolled through other means [27]. The positive effects of using multimedia portals in the informed consent process also remain unclear for socio-economically disadvantaged groups [28].

### Key facilitators to be considered in the implementation of a meta-consent model for LHSs in Quebec

#### Information and transparency

Trust appeared to be the most important element to engage citizens to share their health data for research purposes. A number of previous studies have also identified receiving timely and complete information as a central element to foster trust [6]. People want to know not only about the existence of LHSs but also about its functioning and outputs: ongoing research activities, mechanisms underlying the consent process, research results, and the governance structure. Indeed, for the participants in our focus groups, information was central to the success of the implementation of a meta-consent model for LHSs in Quebec. For our focus group participants, information met several needs.

##### Information to ensure transparency in the processes

Past deficiencies in communication and information on research using people’s data might lead to patients opting out of research projects that could use their data [29]. As a matter of fact, Hill et al. [30] have highlighted that past research suggested that people are generally unaware of research processes and existing safeguards, and that a higher education may increase their acceptability of research using their data without their prior informed consent. Informing citizens about research projects that use their data seemed to have a major influence in maintaining trust and willingness to share their data and to use the consent portal. Furthermore, transparency in data access and data use (explaining which data are being used, who is using them and for what purposes) will not only maintain trust but may also make waiving of consent more acceptable in specific research contexts [30]. With a similar qualitative approach, Hill et al. [30] also have come to the conclusion that their focus group participants became more accepting of the use of medical data without their consent after being given information about selection bias and research processes. In recent years, the Quebec citizens have faced a number of scandals regarding the inappropriate use of their data, including health data [31], banking data [32, 33] and personal data on social networks [34]. The growing number of events surrounding these inappropriate uses makes transparency a challenge but also a cornerstone in building trust regarding the use of health data. More than ever in our society, people are expecting to be told exactly how their data are being used [35].

##### Information regarding the implementation of a web-based portal to allow citizens to express their meta-consent preferences

Public awareness campaigns through diversified information channels, and in particular through primary healthcare professionals, would increase trust and interest regarding the use of the consent portal. Indeed, if people are well informed about the implementation of such a portal by credible sources, they might be more open and inclined to use it.

##### Information regarding the direct impact of research with health data on the improvement of care

Informing citizens about the predicted or potential health care benefits provided by the research project that aims at using their data may improve their understanding and increase their willingness to share. Indeed, in accordance with prior studies [24, 30], our focus group participants were particularly willing to share their health data for research if it was made clear that the purpose was to advance knowledge and improve patient care.

#### Involving healthcare professionals

One of the key elements in the information process regarding the implementation of a web-based meta-consent portal was the involvement of community-based healthcare professionals. These healthcare professionals, especially primary care physicians, but also pharmacists and nurses, were seen as credible and trustful resources. The vast majority of our participants believed that community-based healthcare professionals could play a valuable role in informing people about the consent portal and its function. They could also be primary resource to support the informed decision-making process regarding the use of their health data for research. Again, these results are in line with the literature on similar topics. As mentioned in Kelley et al. [36], trust in the primary care physician seems to be one of the major factors in the acceptability to participate in research within a LHS. A discussion about the general use of health data with primary care physician seems to be valued. According to Kraybill et al. [37], patients with a high level of trust in their physician and in the healthcare system also express a higher level of acceptance for an opt-out model of consent for pragmatic clinical trials comparing standard of care interventions. Also, it will be important to consider whether certain vulnerable populations including patients with cognitive impairment or decline have a lower understanding of a web-based technology and its implications. The resulting ability to give a truly informed consent because of cognitive capacity and/or access to adequate information are areas of concern [37].

#### Establishing close partnerships with recognized institutions

For practical reasons and to gain public trust, a close partnership with a recognized institution was put forth by the participants as a major element for the successful implementation of a meta-consent model in Quebec. The Québec Health Booklet portal, managed by the provincial health insurance organisation (RAMQ) is an online electronic patient record accessible to all Quebec residents to have an integrated view of some health related information captured across hospitals, clinics, private laboratory, etc. [22]. Residents can access the history of their pharmacy medication, their medical imaging reports and receive their laboratory results. For example, currently, COVID-19 test results are made available in the portal. Participants proposed that the two portals (The Québec Health Booklet portal and the meta-consent portal) be integrated for ease of use and comprehension.

#### Establishing default settings deemed acceptable by the society

Adopting new habits takes time [38], and people can be resistant to change (fear of the unknown, misunderstanding about the need for change/when the reason for the change is unclear etc.) [39]. It might take a long time for the population to adopt and use the consent portal. Several participants in our focus groups mentioned that they had no personal interest in using the consent portal (or even more generally in managing their health data for research) as they often trusted the system or had some reservations regarding its use.

Additionally, an advisory group of experts (mostly researchers) consulted to help develop the tools for this study has raised specific concerns regarding the potentially negative impact of the implementation of a meta-consent model (compared to the current models of delegated consent) on research with health data. They considered that such research is already difficult in Quebec because of the current laws, and they were particularly concerned about the potential non-participation or the massive refusal from citizens regarding the use of their health data for research. A massive withdrawal is unlikely based on the survey mentioned previously and the results of the focus group presented here. Nevertheless, because of reservations about the approach, or to the contrary, given a high level of trust in the system (and so assuming that the default settings do not need to be changed), many could choose not to interact with the portal (or other means to make their choices explicit). To avoid blocking the system, defaults meta-consent settings would be a good avenue.

Meta-consent default settings in the meta-consent portal may provide an adequate balance between support of research and social acceptability. Interestingly, the majority of focus group participants were supportive of a broad consent default setting (modifiable or immutable) for certain categories of research projects, for example research projects conducted by public or academic organisations with data used only within Canadian borders, that do not require data with direct identifiers nor genetic information. They also accepted immutable default settings authorizing sharing data pertaining to some specific categories for all participants. They particularly favoured delegating decisions about such default settings to research ethics committees, which they felt best represented their interests.

#### Developing appropriate education tools and public awareness campaigns

Our participants made it clear that educational tools must be developed in order to allow citizens, including those who have no previous experience with research using health data, understand the different characteristics of such research. Educational tools may foster people’s informed consent choices and may increase their willingness to share their data for research. Indeed, in their narrative review, Kalkman et al. [24] showed that a lack of understanding and awareness concerning the use of data was a barrier to data sharing. Awareness campaigns would also essential prior to the implementation of the consent portal in order to build trust and avoid an outcry from citizens. The short videos presented during the focus groups were appreciated and also suggest that similar tools might be helpful. In addition, patients are increasingly exposed to shared, web-based portals centered on care which should help bridge the numerical literacy gap [40–43].

## Limitations

This study is the first in Quebec to elicit people’s attitudes regarding a meta-consent model, with a focus on citizens from various educational backgrounds, and not only frequent users of the healthcare system. Indeed, as this new model of consent shall concern the entire society, it was important to seek the opinion of Quebec citizens in general. However, some specific groups of citizens were most certainly not adequately represented in our limited sample—for example people with mental health disorders. In addition, given its small size, our sample is unlikely to have captured the point of view of Quebec’s first people’s communities and certain minority groups (for example, recent immigrants). Some groups place a higher importance on communal consents and so will require further studies to better structure data access consents. In order to extend our results to these groups, we will need to conduct focus groups specifically adapted for these populations. Finally, due to recruitment difficulties, it was a challenge to include citizens from more remote areas in our sample as well as much younger and older age groups. As a result, their views may be under-represented. Future studies should investigate more specifically the opinion of such populations.

Focus groups are a well-established method to obtain rich qualitative data to adequately portray a complex question. However, focus groups are not exempt of biases, such as the social desirability bias [44], namely the tendency of respondents to answer questions in a manner that will be viewed favorably by others. Interviewers, who did not include the principal investigators, strived at soliciting all point of views, often validated responses, and actively sought alternative opinions to ensure that they were well captured. However, focus groups may nonetheless lead to a form of social consensus. The collective voice which emerges in this context may reflect individuals’ already held opinions, or it may be an active product of the group interactions. It may or may not express the views of all the participants in the group [45]. Nevertheless, we believe our findings point towards essential elements in the first planning steps toward the implementation of a new consent framework in Quebec for the use of people’s health data for research purposes.

The consent preferences of the participants in our focus groups regarding the categories of research projects (results of Fig. 1) should be interpreted with some caution, as the primary objective of this study was to assess the comprehension and acceptability of the meta-consent model itself rather than to identify the characteristics of a research projects that influence most the consent preferences. The characteristics used to define the research categories in our study were chosen based on the literature and through consultation with a panel of experts. No that the meta-consent model itself has now been assessed a suitable approach to support learning health systems (in Quebec), the exact parameters for its implementation will need to be identified, including what characteristics should be used to define the categories.

## Conclusion and questions for future research

In summary, this focus group based qualitative study confirmed the pertinence of the meta-consent model as a socially acceptable alternative (versus the existing legal and ethical framework) to better support LHSs. It pointed to the several key facilitators that should be seriously considered in the implementation phases of a meta-consent portal in the Quebec LHS: transparency and information; support from healthcare professionals; involvement of recognized institutions in the implementation process; default settings deemed acceptable by the society; and a comprehensive approach to public education.

The major findings of this study are intended to guide decision-makers in the next phases of implementation of different strategies to support access to health data in Quebec and to help other jurisdictions in adapting the ethical and legal frameworks to emerging LHSs. In relation to the development of LHSs, several elements need to be taken into consideration:

1. More research is required to determine which level of information should be provided to the citizens to support transparency and informed consent process while avoiding excessive solicitation and anxiety caused by overwhelming information.
2. Although the general meta-consent model was well received and well understood by the participants of this study, the characteristics used, while proposed by the expert committee, were not meant as definitive choices to be implemented “as-is” and were not challenged during the focus groups. Further work is required to identify characteristics that most influence the citizens’ choices of consent type.
3. Further work is also required to determine the meta-consent default settings (modifiable and immutable) that will be put into place. Participants of this study widely desired the involvement of research ethics committees as well as setting up citizen participatory processes for deciding on these.
4. It would be important to explore healthcare professionals’ perceptions about their potential role in the information and meta-consent process. One of the next steps could be to initiate a dialogue with front-line healthcare professional groups on such matters.
5. Finally, in the next phase of the implementation of the consent portal, it would be important to explore and evaluate the possibility of linking it to an existing trusted technological solution such as the Québec Health Booklet portal.

## Supplementary Information

**Additional file 1. Additional methodology information.** The Additional file 1 presents details on the composition of the eight focus groups (Additional Table S1.1), a description of the meta-consent model as we defined it in our study (Additional Tables S1.2, S1.3 and S1.4) as well as the tools used with our focus group participants (Additional Tables S1.5 and S1.6). Additional **Table S1.1**. Focus groups’ composition. Additional **Table S1.2**. Meta-consent model: Set of data content characteristics {X}. Additional **Table S1.3**. Meta-consent model: Set of contextual characteristics {Y}. Additional **Table S1.4**. Meta-consent model: Set of consent choices {Z}. Additional **Table S1.5**. Characteristics defining categories of research projects in our meta-consent model as presented to the focus group participants. Additional **Table S1.6**. Example of predefining categories of research projects for which focus group participants had to express their meta-consent preferences.

**Additional file 2. Additional quotes from participants.** The Additional file 2 presents additional citations from participants regarding reasons for liking or disliking each of the four consent options (Additional Table S2.1) as well as regarding reassuring factors and concerns cited towards each of the eight characteristics of the meta-consent model (Additional Table S2.2). Additional **Table S2.1**. Some reasons cited by our participants for liking or disliking each of the four consent options. Additional **Table S2.2**. Overview of some reassuring factors and concerns cited by our participants regarding each of the eight characteristics of the meta-consent model.

## Data Availability

Parts of the datasets generated and/or analysed during the current study are not publicly available due to obvious re-identification risks from narrative but are available from the corresponding author on reasonable request if the legislation permits it.

## Abbreviations

BCDS: Broad Consent Default Setting
HSD: High School Diploma
LHS: Learning Health System
RAMQ: Régie de l’Assurance Maladie du Québec

## Footnotes

1 Blanket consent refers to the action of an individual to give consent for the use of her/his health data for research without any restrictions [19].

## References

[1] Institute of Medicine. The Learning Healthcare System: Workshop Summary [Internet]. In Olsen L, Aisner D, McGinnis JM, editors. Washington (DC): National Academies Press (US); 2007 [cited 2020 AprIL 23]. (The National Academies Collection: reports funded by National Institutes of Health). http://www.ncbi.nlm.nih.gov/books/NBK53494/. 21452449

[2] Ethier J-F, McGilchrist M, Barton A, Cloutier A-M, Curcin V, Delaney BC (2018). The TRANSFoRm project: experience and lessons learned regarding functional and interoperability requirements to support primary care. Learn Health Syst.

[3] Friedman CP, Rubin JC, Sullivan KJ (2017). Toward an information infrastructure for global health improvement. Yearb Med Inform.

[4] GRIIS. PARS3 [Internet]. GRIIS. 2019 [cited 2020 Feb 4]. https://griis.ca/en/solutions/pars3/.

[5] Cumyn A, Dault R, Barton A, Cloutier AM, Ethier JF. Citizens, research ethics committee members and researchers' attitude toward information and consent for the secondary use of health data: implications for research within learning health systems. J Empir Res Hum Res Ethics. 2021. 10.1177/1556264621992214. 10.1177/1556264621992214 PMC8236664 33710932

[6] Cumyn A, Barton A, Dault R, Cloutier A-M, Jalbert R, Ethier J-F (2019). Informed consent within a learning health system: a scoping review. Learn Health Syst.

[7] Ploug T, Holm S (2016). Meta consent—a flexible solution to the problem of secondary use of health data. Bioethics.

[8] BIP Research. BIP Research [Internet]. 2015 [cited 2020 Mar 2]. https://www.bipresearch.com/.

[9] Gouvernement du Canada SC. Tableau A4.1 Description des niveaux de compétence en littératie du Programme pour l’évaluation internationale des compétences des adultes, 2012 [Internet]. 2016 [cited 2020 June 8]. https://www150.statcan.gc.ca/n1/pub/89-657-x/2016001/tbl/tblA4-1-fra.htm.

[10] Alphare. Alphare - Centre d’alphabétisation [Internet]. Alphare - Centre d’alphabétisation populaire de Beauce Alphare - Centre d’alphabétisation populaire de beauce. 2015 [cited 2020 June 9]. http://www.alphare.ca/.

[11] Yamaska Literacy Council. Yamaska Literacy Council [Internet]. Yamaska Literacy Council. [cited 2020 June 9]. https://yamaskaliteracy.wordpress.com/.

[12] Nyumba TO, Wilson K, Derrick CJ, Mukherjee N (2018). The use of focus group discussion methodology: insights from two decades of application in conservation. Methods Ecol Evol.

[13] Institut de la Statistique du Québec. Zoom Santé—Novembre 2007. 2007;4.

[14] GRIIS. Vimeo - GRIIS [Internet]. Vimeo. 2020 [cited 2020 Apr 23]. https://vimeo.com/griis.

[15] Thaler RH, Sunstein CR (2009). Nudge: improving decisions about health, wealth, and happiness.

[16] Barton A, Grüne-Yanoff T (2015). From libertarian paternalism to nudging—and beyond. Rev Philos Psychol.

[17] Seppälä S, Ruttenberg A, Schreiber Y, Smith B (2016). Definitions in ontologies. Cah Lexicol.

[18] Ware JE, Gandek B (1998). Overview of the SF-36 Health Survey and the International Quality of Life Assessment (IQOLA) Project. J Clin Epidemiol.

[19] Wendler D (2013). Broad versus blanket consent for research with human biological samples. Hastings Cent Rep.

[20] Steele C. What is the digital divide? [Internet]. Digital Divide Council. 2019 [cited 2020 June 11]. http://www.digitaldividecouncil.com/what-is-the-digital-divide/.

[21] Gouvernement du Québec. Accueil | Régie de l’assurance maladie du Québec (RAMQ) [Internet]. 2020 [cited 2020 Nov 23]. https://www.ramq.gouv.qc.ca/fr.

[22] Gouvernement du Québec. The Québec Health Booklet—Home [Internet]. 2020 [cited 2020 June 12]. https://carnetsante.gouv.qc.ca/portail?Langue=en.

[23] Howe N, Giles E, Newbury-Birch D, McColl E (2018). Systematic review of participants’ attitudes towards data sharing: a thematic synthesis. J Health Serv Res Policy.

[24] Kalkman S, Delden J van, Banerjee A, Tyl B, Mostert M, Thiel G van. Patients’ and public views and attitudes towards the sharing of health data for research: a narrative review of the empirical evidence. J Med Ethics [Internet]. 2019 Nov 12 [cited 2020 May 7]. http://jme.bmj.com/content/early/2019/11/11/medethics-2019-105651. 10.1136/medethics-2019-105651 PMC8717474 31719155

[25] Ploug T, Holm S (2017). Eliciting meta consent for future secondary research use of health data using a smartphone application—a proof of concept study in the Danish population. BMC Med Ethics.

[26] Budin-Ljøsne I, Teare HJA, Kaye J, Beck S, Bentzen HB, Caenazzo L (2017). Dynamic Consent: a potential solution to some of the challenges of modern biomedical research. BMC Med Ethics.

[27] Boutin NT, Mathieu K, Hoffnagle AG, Allen NL, Castro VM, Morash M (2016). Implementation of electronic consent at a Biobank: an opportunity for precision medicine research. J Pers Med.

[28] Nishimura A, Carey J, Erwin PJ, Tilburt JC, Murad MH, McCormick JB (2013). Improving understanding in the research informed consent process: a systematic review of 54 interventions tested in randomized control trials. BMC Med Ethics.

[29] Hays R, Daker-White G (2015). The care.data consensus? A qualitative analysis of opinions expressed on Twitter. BMC Public Health.

[30] Hill EM, Turner EL, Martin RM, Donovan JL (2013). “Let’s get the best quality research we can”: public awareness and acceptance of consent to use existing data in health research: a systematic review and qualitative study. BMC Med Res Methodol.

[31] La Presse. Dossiers médicaux à vendre [Internet]. La Presse. 2018 [cited 2020 May 7]. http://www.lapresse.ca/actualites/sante/201803/02/01-5155859-dossiers-medicaux-a-vendre.php.

[32] La Presse. 2,9 millions de membres de Desjardins victimes d’une faille de sécurité [Internet]. L’actualité. 2019 [cited 2020 June 12]. https://lactualite.com/actualites/29-millions-de-membres-de-desjardins-victimes-dune-faille-de-securite/.

[33] Le Devoir. Fuite de données massive chez Capital One [Internet]. Le Devoir. 2019 [cited 2020 June 12]. https://www.ledevoir.com/societe/559692/fuite-de-donnees-massive-chez-capital-one.

[34] Le Journal de Montréal. Tout ce que vous devez savoir pour comprendre le scandale Facebook-Cambridge Analytica|JDM [Internet]. 2018 [cited 2020 June 12]. https://www.journaldemontreal.com/2018/03/20/scandale-facebook-cambridge-analytica-tout-ce-que-vous-avez-manque.

[35] Häikiö J, Yli-Kauhaluoma S, Pikkarainen M, Iivari M, Koivumäki T (2020). Expectations to data: perspectives of service providers and users of future health and wellness services. Health Technol.

[36] Kelley M, James C, Alessi Kraft S, Korngiebel D, Wijangco I, Rosenthal E (2015). Patient perspectives on the learning health system: the importance of trust and shared decision making. Am J Bioeth.

[37] Kraybill A, Dember LM, Joffe S, Karlawish J, Ellenberg SS, Madden V (2016). Patient and physician views about protocolized dialysis treatment in randomized trials and clinical care. AJOB Empir Bioeth.

[38] Lally P, van Jaarsveld CHM, Potts HWW, Wardle J (2010). How are habits formed: Modelling habit formation in the real world. Eur J Soc Psychol.

[39] Kanter RM. Ten reasons people resist change. Harvard Business Review [Internet]. 2012 Sep 25 [cited 2020 Nov 25]. https://hbr.org/2012/09/ten-reasons-people-resist-chang.

[40] Jameie S, Haybar H, Aslani A, Saadat M (2019). Development and usability evaluation of web-based telerehabilitation platform for patients after myocardial infarction. Stud Health Technol Inform.

[41] Bernhard G, Mahler C, Seidling HM, Stützle M, Ose D, Baudendistel I (2018). Developing a shared patient-centered, web-based medication platform for type 2 diabetes patients and their health care providers: qualitative study on user requirements. J Med Internet Res.

[42] Hensel JM, Shaw J, Ivers NM, Desveaux L, Vigod SN, Cohen A (2019). A web-based mental health platform for individuals seeking specialized mental health care services: multicenter pragmatic randomized controlled trial. J Med Internet Res.

[43] Klement MR, Rondon AJ, McEntee RM, Greenky MR, Austin MS (2019). Web-based, self-directed physical therapy after total knee arthroplasty is safe and effective for most, but not all, patients. J Arthroplasty.

[44] Social desirability. In: Encyclopedia of survey research methods [Internet]. Thousand Oaks: Sage Publications, Inc.; 2008 [cited 2020 Jun 12]. http://methods.sagepub.com/reference/encyclopedia-of-survey-research-methods/n537.xml.

[45] Smithson J (2000). Using and analysing focus groups: Limitations and possibilities. Int J Soc Res Methodol.

