# Supplementary material for: Meta-consent for the secondary use of health data within a learning health system: a qualitative study of the public’s perspective
Source: BMC Med Ethics. 2021 Jun 29;22:81. doi: 10.1186/s12910-021-00647-x (PMC8240433; doi:10.1186/s12910-021-00647-x)
Supplement: Supplementary file 1 — Additional file 1. Additional methodology information. The Additional file 1 presents details on the composition of the eight focus groups (Additional Table S1.1), a description of the meta-consent model as we defined it in our study (Additional Tables S1.2, S1.3 and S1.4) as well as the tools used with our focus group participants (Additional Tables S1.5 and S1.6). Additional Table S1.1. Focus groups’ composition. Additional Table S1.2. Meta-consent model: Set of data content characteristics {X}. Additional Table S1.3. Meta-consent model: Set of contextual characteristics {Y}. Additional Table S1.4. Meta-consent model: Set of consent choices {Z}. Additional Table S1.5. Characteristics defining categories of research projects in our meta-consent model as presented to the focus group participants. Additional Table S1.6. Example of predefining categories of research projects for which focus group participants had to express their meta-consent preferences. [file 12910_2021_647_MOESM1_ESM.docx]

**Additional file 1. Additional methodology information**

**Additional Table S1.1. Focus groups’ composition**

| Focus Group | Education* | Language | Location | Participants | Age range | Gender |
| --- | --- | --- | --- | --- | --- | --- |
| 1 | No HSD | French | Sherbrooke | 7 | 30 – 67 | M: 6  F: 1 |
| 2 | ≥ HSD | French | Sherbrooke | 11 | 20 – 70 | M: 4  F: 7 |
| 3 | No HSD | English | Montreal | 6 | 30 – 58 | M: 4  F: 2 |
| 4 | ≥ HSD | English | Montreal | 8 | 34 – 60 | M: 3  F: 5 |
| 5 | No HSD | French | Rimouski | 8 | 27 – 66 | M: 4  F: 4 |
| 6 | ≥ HSD | French | Rimouski | 8 | 27 – 66 | M: 3  F: 5 |
| 7 | No HSD | French | Quebec City | 8 | 31 – 70 | M: 3  F: 5 |
| 8 | ≥ HSD | French | Quebec City | 7 | 24 – 66 | M: 5  F: 3 |
| HSD: High School Diploma.  *No HSD: participants who have no high school diploma nor equivalent; ≥ HSD: participants who have at least a high school diploma or equivalent (including a diploma of collegial studies, a university diploma or equivalent). | | | | | | |

**Additional Table S1.2. Meta-consent model: Set of data content characteristics {X}**

| **Data identification character** | | **Genetic data usage** | |
| --- | --- | --- | --- |
| *With direct identifiers* | *Without direct identifiers* | *With genetic information* | *Without genetic information* |
| The data include at least one direct identifier. | The data exclude any direct identifier. | The data include at least one genetic information. | The data exclude any type of genetic information. |
| Examples of direct identifier: the name, the personal health plan number, the social insurance number (1). | Examples of non-direct identifier: the first three digits of the postal code, year of birth. | Genetic data results from the analysis of specific genes obtained from a biological sample (blood, hair, skin, etc.) (2).  Genetic data can reveal information about susceptibility to diseases and physical conditions (3) and can help to understand hereditary diseases as well as the response of an individual to drugs and treatments (2). | Example of non-genetic data: weight, blood pressure measurement, lung X-ray. |

**Additional Table S1.3. Meta-consent model: Set of contextual characteristics {Y}**

| **Type of organisation who has access to the data** | | **Where could the data be used** | |
| --- | --- | --- | --- |
| *Academic or public organisation* | *Private organisation with commercial goals* | *Only within Canadian borders* | *International* |
| A public or academic organisation has access to the data. | A private organisation with commercial goals has access to the data. | Data are being used at a national scale only (within Canadian jurisdiction). | Data are being used at an international scale (within Canadian jurisdiction and those of at least another country). |
| A public organisation refers to an organisation or a company that is under the jurisdiction of the state or public authorities (4).  An academic organisation refers to an educational institution dedicated to higher education and research, which grants academic degrees (4).  For example, data are accessed by governments, hospitals, universities, research institutes, research centers, or non-profit organisations like foundations. | A private organisation with commercial goals refers to an organisation or a company that belong to private individuals or legal entities rather than the state or public authorities, and for which there are commercial goals.  For example, data are accessed by a pharmaceutical company who specializes in the development of new drugs or by a company who develops medical devices. | For example, data are being used by researchers from two Canadiens universities (e.g. McGill University and The University of British Columbia). | For example, data are being used in collaboration by researchers from a university in Canada and by researchers from a university in Japan. |

**Additional Table S1.4. Meta-consent model: Set of consent choices {Z}**

| **Broad approval** | **Specific opt-out** | **Specific opt-in** | **Broad refusal** |
| --- | --- | --- | --- |
| For a given category of research projects, **I authorize** the use of my health data for all research projects from this category. | For a given category of research projects, **I want to be asked** for my consent for each new research project from this category that would like to use my data.  From the moment I am informed of the new research project, I have a given period of time (e.g. 3 months) to refuse access to my data for this project. If I have not taken action by the end of this period, my data **will be used** for the said project. | For a given category of research projects, **I want to be asked** for my consent for each new research project from this category that would like to use my data.  From the moment I am informed of the new research project, I have a given period of time (e.g. 3 months) to authorize access to my data for this project. If I have not taken action by the end of this period, my data **will not be used** for the said project. | For a given category of research projects, **I refuse** the use of my health data for all research projects from this category. |

**Additional Table S1.5. Characteristics defining categories of research projects in our meta-consent model as presented to the focus group participants**

| **Data identification character** | |
| --- | --- |
| **With direct identifiers** 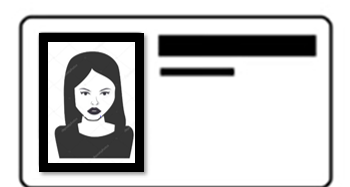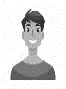 e.g., first and last name, personal  health plan number,  cell phone number. | **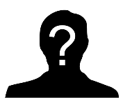Without direct identifiers**  e.g., year of birth, postal code. |
| **Genetic data usage** | |
| **With genetic information**  e.g., analysis of genes responsible  for ​hereditary diseases from a  blood sample. | **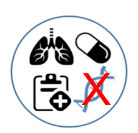Without genetic information**  e.g., ​ weight, blood pressure  measure, lung X-ray. |
| **Type of organisation who has access to the data** | |
| **Academic or public organisation**​  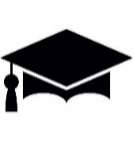  e.g., universities, hospitals. | **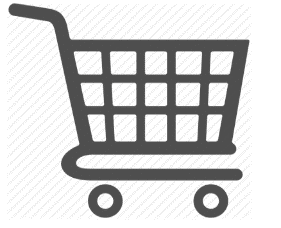Private organisation with commercial goals**  e.g., a pharmaceutical company  developing a new medication or  medical device. |
| **Where could the data be used** | |
| **Only within Canadian borders**  **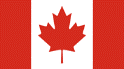**e.g., researchers from two Canadian  universities (McGill University and  The University of British Colombia). | **International** 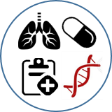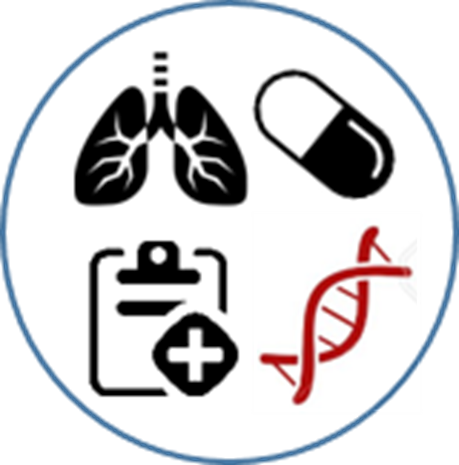 **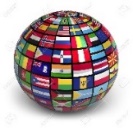**  e.g., researchers from a university  in Canada and from a university  in Japan. |

**Additional Table S1.6. Example of predefining categories of research projects for which focus group participants had to express their meta-consent preferences**

| **Category A** | **Category B** | **Category C** |
| --- | --- | --- |
| 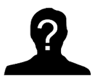 | 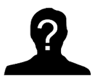 | 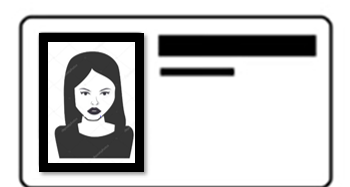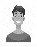 |
| 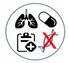 | 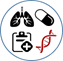 | 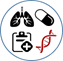 |
| 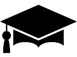 | 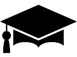 | 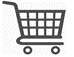 |
| 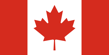 | 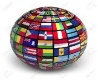 | 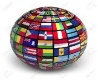 |
| **Consent choice?** | **Consent choice?** | **Consent choice?** |

**Consent choices (using stickers):**

| **Broad approval** | **Specific opt-out** |
| --- | --- |
| **Specific opt-in** | **Broad refusal** |

**References**

1. Instituts de recherche en santé du Canada, Conseil de recherches en sciences humaines du Canada, Conseil de recherches en sciences naturelles et en génie du Canada. EPTC 2 points saillants des modifications. 2014.

2. World Health Organization, éditeur. Genomics and world health: report of the Advisory Committee on Health Research. Geneva: World Health Organization; 2002. 241 p.

3. Anderson NB, Bulatao RA, Cohen B, National Research Council (US) Panel on Race E. Genetic Factors in Ethnic Disparities in Health [Internet]. Critical Perspectives on Racial and Ethnic Differences in Health in Late Life. National Academies Press (US); 2004 [cité 29 oct 2020]. Disponible sur: https://www.ncbi.nlm.nih.gov/books/NBK25517/

4. Gouvernement du Québec. Thésaurus de l’activité gouvernementale [Internet]. 2020 [cité 29 oct 2020]. Disponible sur: http://www.thesaurus.gouv.qc.ca/tag/accueil.do;jsessionid=0AA5C2E9BF3B12C3E0DFDE038FAFB893
