# Supplementary material for: Meta-consent for the secondary use of health data within a learning health system: a qualitative study of the public’s perspective
Source: BMC Med Ethics. 2021 Jun 29;22:81. doi: 10.1186/s12910-021-00647-x (PMC8240433; doi:10.1186/s12910-021-00647-x)
Supplement: Supplementary file 2 — Additional file 2. Additional quotes from participants. The Additional file 2 presents additional citations from participants regarding reasons for liking or disliking each of the four consent options (Additional Table S2.1) as well as regarding reassuring factors and concerns cited towards each of the eight characteristics of the meta-consent model (Additional Table S2.2). Additional Table S2.1. Some reasons cited by our participants for liking or disliking each of the four consent options. Additional Table S2.2. Overview of some reassuring factors and concerns cited by our participants regarding each of the eight characteristics of the meta-consent model. [file 12910_2021_647_MOESM2_ESM.docx]

**Additional file 2. Additional quotes from participants**

**Additional Table S2.1. Some reasons cited by our participants for liking or disliking each of the four consent options**

| **Broad approval** | **Specific Opt-Out** | **Specific Opt-In** | **Broad refusal** |
| --- | --- | --- | --- |
| (+) “It’s simply going to help with research. […]. If it’s helpful for me, good, but if it’s to help other people, well, it’s even better.”  (+) “The more data they have, the better... I would be a nice case, let's say. They would have a lot of information.” | (+) “I need to know why and who is using my data before they want to use it.”  (+) “I will accept if I am informed more in advance. I just don’t want to block the research only because I didn’t answer the call.”  (+) “I just want to be in control and see what it is. So, probably most of the time, it’s going to be yes, but I just want to have the choice to say it.”  (-) “You can be bombarded with these emails every day? ‘We need you to do a research’ You have to answer every time?” | (+) “I would definitely choose the specific opt-in. If I don’t answer, I refuse. Because the opt-out makes me very nervous.”  (+) “I want to know where it[data]’s going. And if I don’t like where it’s going, what they’re using it for, then forget it. If I forget to answer, then it’s a refusal.”  (-) “The lack of understanding, the fear of the unknown… there are people who will imagine a lot of things, while the reality is different. So, with the specific opt-in, there will be a lot of people who will say ‘no’ and it will have a negative impact on the whole population.” | Data with direct identifiers****  Private organizations with commercial goals***  International**  Data with genetic information* |
| Notes:  ****Characteristic within a given category of research projects most cited by the participants to justify the choice of broad refusal, ***Second most cited characteristic, **Third most cited characteristic, *Fourth most cited characteristic. | | | |

**Additional Table S2.2. Overview of some reassuring factors and concerns cited by our participants regarding each of the eight characteristics of the meta-consent model**

| **Data identification character** | |
| --- | --- |
| **With direct identifiers** | **Without direct identifiers** |
| (+) “If there is no Social Insurance Number, I have no problem, go for it.”  (+) “Personally, I think I would rather do a study where they want to know who I am. I want to be involved. I want to know what they are doing. You want to know who I am, I want to know you.”  (+) “I have Crohn’s disease and if they do something like this, a study group on Crohn, and they need participants, I’ll be open to that.”  (+) “I’m opening a debate here, but if you have lung cancer and Pfizer finds a drug and researches it, and says, “well Marco* has lung cancer and I’m able to cure him, and I have his phone number, I’m able to call him.”  (-) “Yes, I mean direct identifiers made me very nervous. I also don’t quite understand the value of giving you my information. I just don’t like it. It just doesn’t make too much sense to me.”  (-) “I don’t want to be contacted.”  (-) “Once we accept their research, especially if they have direct identifiers...What’s to stop them from taking that and selling it for commercial purposes?” | (+) “For me, the fact that my name does not appear makes it OK.”  (+) “I accept, because it can be useful for some progress in the field of research, and my name does not appear anywhere.” |
| **Genetic data usage** | |
| **Without genetic information** | **With genetic information** |
| (-) “I wouldn’t be interested in the research if they [researchers] don’t need these data [genetic data].”  (-) “What’s the point of a research without genetic data?” | (+) “That [genetic data] is important in research.”  (-) “Genetic data is too much information.” |
| **Type of organisation who has access to the data** | |
| **Academic or public organisation** | **Private organisation with commercial goals** |
| (+) “The university researchers are above all interested in health, which is important for me.”  (-) “Academic and public organizations, their security is usually a lot weaker than others.” | (+) “With private research, yes, we know that there is always been flaws and there will always be. But we know that even if it’s private, it doesn’t mean that they won’t find anything, it means that they have the possibility to find something very interesting. They can also help people with their research.”  (+) “Well, I would say it’s limited because, for example, I’m totally against anything like that commercialization. But at the same time, if they can build a good wheelchair, they will need that data too.”  (-) “I just want to make sure they (private organizations) are not going to try to sell me something that I’m not interested in.”  (-) “In the private sector, sometimes they have more freedom, they can do what they want with the data. They might contact the insurance companies, sell the data...We don’t know.” |
| **Where could the data be used** | |
| **Only within Canadian borders** | **International** |
| (+) “Like I said, I have faith in our system here.” | (+) “There are good scientists elsewhere, and maybe two heads are better than one. If they gather the data and discover things, that would be good.”  (+) International in this scenario doesn’t really bother me because the advances for my son’s condition came from international research.”  (+) “I’m relatively very open to it... Sharing with others and receiving from others as well. I think we can learn a lot and we can also demonstrate our knowledge which is very interesting.”  (-) “I don’t know what their health system represents and what precautions they have on their side to protect us. So that’s why I would like to know which type of project first. Prior to letting, let’s say, a foreign country uses it.”  (-) “It’s the word ‘international’ that scares me. I don’t know, when my data is going out, I’m not comfortable.”  (-) “Because it’s ‘international’, we don’t have any control, and unfortunately, other countries don’t value the information like we do.” |
| Note:  *The participant’s first name has been modified to ensure confidentiality. | |
